# Multiomic Approaches Reveal Hormonal Modulation and Nitrogen Uptake and Assimilation in the Initial Growth of Maize Inoculated with *Herbaspirillum seropedicae*

**DOI:** 10.3390/plants12010048

**Published:** 2022-12-22

**Authors:** Luiz Eduardo Souza da Silva Irineu, Cleiton de Paula Soares, Tatiane Sanches Soares, Felipe Astolpho de Almeida, Fabrício Almeida-Silva, Rajesh Kumar Gazara, Carlos Henrique Salvino Gadelha Meneses, Luciano Pasqualoto Canellas, Vanildo Silveira, Thiago Motta Venancio, Fabio Lopes Olivares

**Affiliations:** 1 Laboratório de Biologia Celular e Tecidual, Centro de Biociências e Biotecnologia, Universidade Estadual do Norte Fluminense Darcy Ribeiro, Campos dos Goytacazes 28013-602, Rio de Janeiro, Brazil; 2 Instituto Federal de Roraima, Campus Novo Paraíso, Caracaraí 69.365-000, Roraima, Brazil; 3 Institute de Química, Universidade de São Paulo, São Paulo 05508-900, São Paulo, Brazil; 4 Laboratório de Biotecnologia, Centro de Biociências e Biotecnologia, Universidade Estadual do Norte Fluminense Darcy Ribeiro, Campos dos Goytacazes 28013-602, Rio de Janeiro, Brazil; 5 VIB-UGent Center for Plant Systems Biology, Ghent University, UGENT, 9000 Ghent, Belgium; 6 Laboratório de Química e Função de Proteínas e Peptídeos, Centro de Biociências e Biotecnologia, Universidade Estadual do Norte Fluminense Darcy Ribeiro, Campos dos Goytacazes 28013-602, Rio de Janeiro, Brazil; 7 Departamento de Biologia, Centro de Ciências da Saúde e Biológica, Universidade Estadual da Paraíba, Campina Grande 58429-500, Paraíba, Brazil; 8 Núcleo de Desenvolvimento de Insumos Biológicos para a Agricultura, Universidade Estadual do Norte Fluminense Darcy Ribeiro, Campos dos Goytacazes 28013-602, Rio de Janeiro, Brazil

**Keywords:** endophytic bacteria, biostimulation, *Herbaspirillum* spp., diazotrophs, plant-growth promotion, biofertilizer

## Abstract

*Herbaspirillum seropedicae* is an endophytic bacterium that can fix nitrogen and synthesize phytohormones, which can lead to a plant growth-promoting effect when used as a microbial inoculant. Studies focused on mechanisms of action are crucial for a better understanding of the bacteria-plant interaction and optimization of plant growth-promoting response. This work aims to understand the underlined mechanisms responsible for the early stimulatory growth effects of *H. seropedicae* inoculation in maize. To perform these studies, we combined transcriptomic and proteomic approaches with physiological analysis. The results obtained eight days after inoculation (d.a.i) showed increased root biomass (233 and 253%) and shoot biomass (249 and 264%), respectively, for the fresh and dry mass of maize-inoculated seedlings and increased green content and development. Omics data analysis, before a positive biostimulation phenotype (5 d.a.i.) revealed that inoculation increases N-uptake and N-assimilation machinery through differentially expressed nitrate transporters and amino acid pathways, as well carbon/nitrogen metabolism integration by the tricarboxylic acid cycle and the polyamine pathway. Additionally, phytohormone levels of root and shoot tissues increased in bacterium-inoculated-maize plants, leading to feedback regulation by the ubiquitin-proteasome system. The early biostimulatory effect of *H. seropedicae* partially results from hormonal modulation coupled with efficient nutrient uptake-assimilation and a boost in primary anabolic metabolism of carbon–nitrogen integrative pathways.

## 1. Introduction

Brazilian agricultural production has grown so much that it stands out for participating in the world’s agricultural production. However, the increased crop area and productivity demand higher consumption of fertilizers and pesticides. As a result, Brazil’s demand for nitrogen (N) and phosphate (P) fertilizers is constantly growing [1,2]. There is a substantial monetary and energy expenditure on nitrogenous fertilizers’ synthesis, storage, transport, and distribution [3]. In the P-fertilizers’ case, the rock phosphate world deposits could be drastically diminished in this century [2]. Additionally, there is a low plant recovery of N-fertilizers applied in agroecosystems with estimated losses of 50 to 70% [1], which results in local contamination of soil and water bodies [1] and global negative impact by atmospheric emission of greenhouse gases, such as nitrous oxide [2,4].

Using microbial inoculants with biofertilizer and biostimulant properties emerges as an alternative to replace or complement synthetic fertilizers to promote plant growth and protection and reduce the environmental impacts of industrial agriculture practices [5]. Furthermore, bio-based agricultural inputs and technologies represent a tool for increasing country sovereignty in the face of the world’s challenges of producing food, fiber, and energy. Furthermore, bioinoculants are aligned with the United Nations Sustainable Development Goal (SDG) number 2, which proposes ending hunger, achieving food security, increasing nutrition, and promoting sustainable agriculture (https://sdgs.un.org/ goals/goal2, accessed on 28 September 2022).

*Herbaspirillum seropedicae* is a nitrogen-fixing bacterium of the β-proteobacteria subclass that is preferably associated with grasses of great economic and food importance, such as maize, sugar cane, rice, sorghum, wheat and forage grasses [6,7]. The bacteria survive poorly in soil but can be found endophytically associated with different plant species’ roots, stems and leaves [6,7,8,9,10]. *H. seropedicae* adheres plant surfaces as epiphytes or migrates to apoplastic compartments of the plant body, colonizing intercellular spaces and xylem lumen as endophytes [9,10]. When associated with the plant host, the bacteria can promote biological nitrogen fixation (BNF), helping to supply the N-demand of plants [11,12,13,14]. In addition, *H. seropedicae* can also produce growth regulators such as auxins and gibberellins [15,16,17] and solubilize inorganic phosphorus [18]. According to genomic sequencing, *H. seropedicae* has genes for four possible production pathways for indole-acetic acid (IAA), also known as the phytohormone auxin [19].

The use of beneficial bacteria as microbial inoculants for grasses in agroecosystems has constantly increased in the last decade, with more than ten million doses sold and occupancy of 12% of the Brazilian market of inoculants for plant growth promotion in 2019 [20]. The maize crop field is dominated by *Azospirillum brasilense* strain Ab-V5 and Ab-V6 commercial inoculants, but there is space for more diverse and efficient bio-products. Brazil is the second larger exporter of maize in the world, with around 16 million hectares of cultivated area [21]. Selected strains of *H. seropedicae* had shown a promising effect in promoting plant growth and protection, being a potential candidate for microbial formulation in maize and other grasses such as grass pastures [22,23,24,25,26,27].

Overall, plant growth mechanisms and effective bio-products of bacterium-plant host interactions are still under debate from a scientific and technological perspective. Herein, we combined omics and physiological analysis to understand the biostimulation effects of *H. seropedicae* inoculation in the early stage of development of maize plantlets, without relying on chemical fertilizers.

## 2. Results

### 2.1. H. seropedicae Association and Plant Growth Promotion

**Figure 1:**
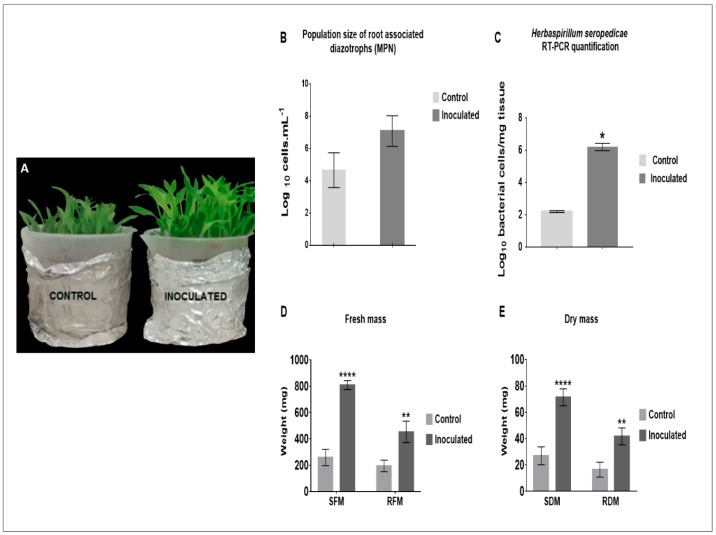
(**A**) Plantlet growth after 8 days after inoculation (d.a.i.) of 2 × 10^7^ cells mL^−1^ of *H. seropedicae* strain HRC54 on maize variety Dekalb 7815. (**B**) The population size of root-associated diazotrophs by Most Probable Number (MPN) at five d.a.i. (**C**) The number of bacterial cells was validated by qPCR. Estimation considered the copy number of the 16S rDNA gene of *H. seropedicae*, Avogadro’s number and the product mass of the amplification. (**D**) Increase in the shoot fresh mass (SFM) and root fresh mass (RFM). (**E**) Increase in the shoot dry mass (SDM) and root dry mass (RDM). Bars marked by an asterisk indicate significant difference between means in the inoculated and control treatments (* p value < 0.05; ** *p* value < 0.01; **** *p*-value < 0.0001). Mean bars with the standard deviation (**B**,**C**: n = 3; D-E: n = 8).

The biostimulation effect of *H. seropedicae* at 8 days after inoculation (d.a.i) was observed in inoculated plantlets (Figure 1A), and counting diazotrophic bacteria proved the effective bacteria-plant association through the most probable number (MPN) technique, that showed their occurrence in the control plants at 4.5 × 10^4^ cells.mL^−1^, and inoculated plants at 1.2 × 10^7^ cells.mL^−1^ at 5 d.a.i. (Figure 1B). The confirmation of *H. seropedicae* in the roots was assessed by conventional PCR amplification using specific primers for the 16S rDNA of *H. seropedicae* (Figure 1C). DNA was extracted from the inoculum and dilutions were used to generate a standard curve ranging from 10^−1^ to 10^−5^ ng.μL^−1^ of DNA. The efficiency of the primer was approximately 98% (Figure S1). The number of bacterial cells in the inoculated maize roots was 10^6^, while the non-inoculated plants presented 10^2^. mg tissue-1 (Figure 1C). The efficiency of the primer was approximately 98% (Table S3).

The biostimulation effect of bacterial association was observed in the inoculated seedlings, where 249.4% was observed in the fresh biomass and 264% in the dry biomass of the shoots (Figure 1D). In addition, growth stimulation was observed in the roots inoculated, with an increase of 233% in fresh biomass and 253% in dry biomass compared with the control (Figure 1D,E).

**Figure 2:**
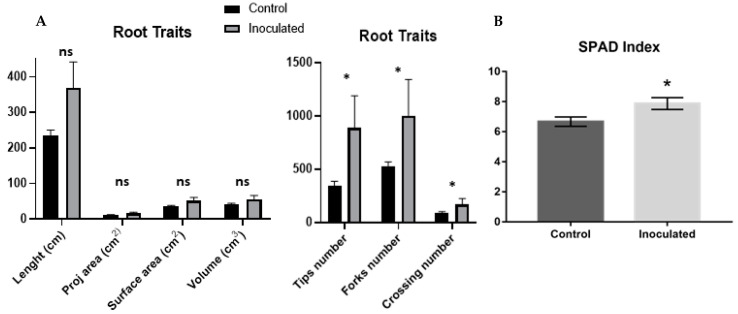
(**A**) WinRhizo image analysis results. (**B**) Green index analysis by SPAD of Maize leaves inoculated and uninoculated with *H. seropedicae*. (±) represent the standard error (n = 5). Means followed by “ns” do not differ in the inoculation condition. Means marked by asterisk differ in the inoculation condition (* *p* value < 0.05). Bars represent the standard error (n = 5).

Image analysis of the root system did not show differences statistically; however, an increase in the values of the inoculated roots could be observed, in addition to the statistical differences in the number of root tips, forks and crossings (Figure 2A).

Quantification of photosynthetic pigments measured by SPAD demonstrated that maize plants inoculated with *H. seropedicae* have an increased green index compared to uninoculated plants. (Figure 2B).

### 2.2. Multiomics Integrative Analysis

RNA-seq was performed using three independent biological replicates (using a mixer of three plants per replicate) of the maize root for each treatment (control and inoculated with *H. seropedicae*), generating six libraries. Control and inoculated maize root samples generated 156.04 and 81.80 million uniquely mapped reads, respectively, uniquely mapped in the maize genome. Statistics on RNA-seq reads are depicted in Table 1. The genes that showed FDR = 0.05 were considered responsive to the inoculation of *H. seropedicae*. We identified 4501 differential expressed genes (DEGs) through the DESeq2 analysis, while edgeR analysis permitted the identification of 5268 DEGs. We commonly identified 4585 DEGs through bioinformatics tools (Table S1).

Proteomic analysis was performed using three independent biological replicates (using a mixture of 5 plants to compose a replicate), generating a total of 240 gene products of maize root in each experimental condition (control and inoculated with *H. seropedicae*), with statistical significance (*p* < 0.05). In addition, the biological replicates demonstrated a high level of correlation (r^2^ > 0.9311) (Table S2).

In summary, 91 proteins were down-accumulated, and 132 proteins had their expression up-accumulated. Interestingly, eleven proteins appeared to be expressed only in control plants, while six other proteins appeared only in plants treated with *H. seropedicae*.

**Figure 3:**
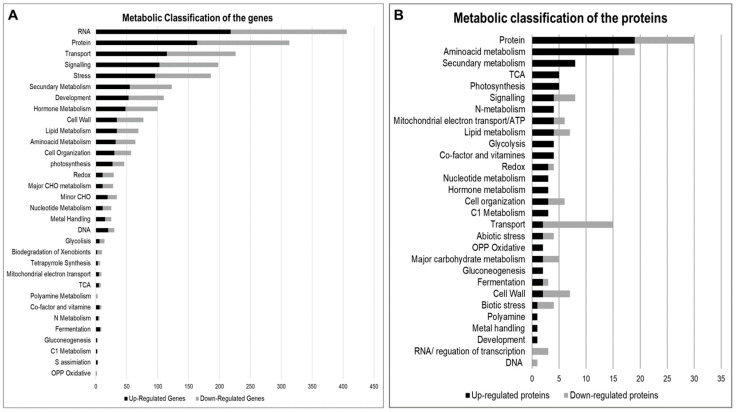
Metabolic functional classification of differentially expressed genes (DEGs) (**A**) and differentially accumulated proteins (DAPs) (**B**) in Maize inoculated with *H. seropedicae*. Legend: CHO (carbohydrate), TCA (tricarboxylic acid), C1 (one-carbon), OPP (oxidative pentose phosphate).

To access the functional classification of gene products, all the modulated genes and proteins were classified according to MapMan, and 3297 DEGs and 191 differentially accumulated proteins (DAPs) were matched (Figure 3). In addition, a total of 1151 genes and 23 proteins were matched to products with unknown functions.

**Figure 4:**
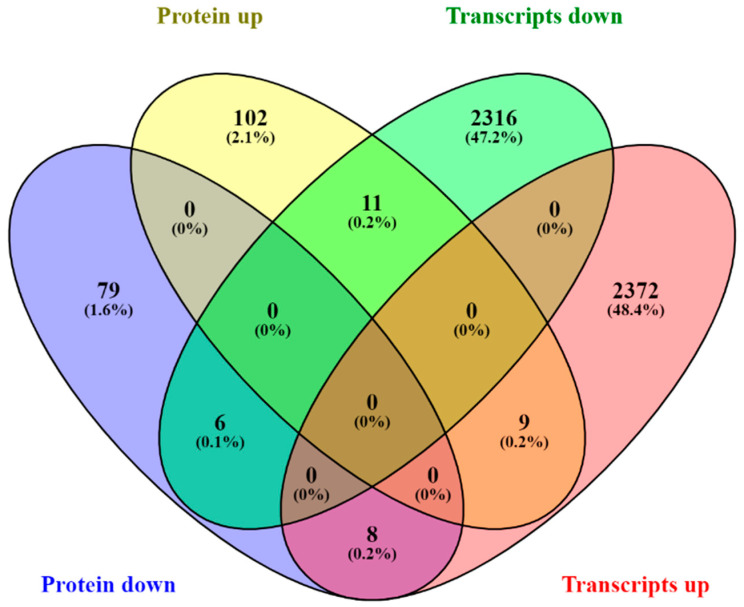
Venn diagram of the correlation of transcripts and proteins of maize roots inoculated or uninoculated with *H. seropedicae* strain HRC54.

We integrated the differential expression in the transcriptome and proteome, getting a slight overlap at the gene level (only 0.7%), in order to investigate whether the changes in protein abundance correlated with those at the corresponding mRNA level (Figure 4).

#### 2.2.1. RNA Transcription

**Figure 5:**
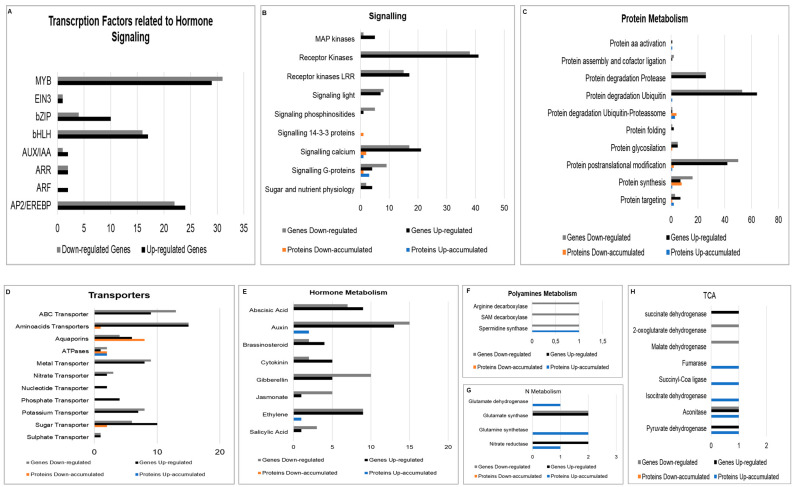
Mapman visualization of differentially expressed genes (DEGs) and differentially accumulated proteins (DAPs) of diverse regulatory processes (**A**), signaling (**B**), metabolic pathways (**C)**, transport (**D**), hormone (**E**), polyamine (**F**), nitrogen (**G**) metabolism and TCA pathway (**H**) of Maize inoculated by *H. seropedicae*. Legend: MAPK (mitogen-activated protein kinases), IAA (indol-3-acid-acetic), ABA (abscisic acid), GA (gibberellin), BA (brassinosteroids) and SA (salicylic acid), SAM (S-adenosylmethionine), TCA (tricarboxylic acid cycle).

RNA classification had a high number of DEGs represented. A total of 406 had 218 upregulated and 188 downregulated (Figure 5). We can find some genes related to RNA processing in this metabolism classification. However, the central part is about transcription factors (TF) related to hormone transduction signals, such as APETALA2/ethylene-responsive element-binding protein family (AP2/EREBP), signalization protein ethylene-insensitive3 (EIN3), auxin response factor (ARF), protein repressor of the auxin synthesis auxin/indol acetic acid (AUX/IAA), receptor of a signal of cytokinin Arabidopsis response regulator (ARR), TFs basic leucine zipper domain (bZIP), TFs basic helix-loop-helix family (bHLH) and ABA-dependent TF myeloblastosis oncogene (MYB) (Figure 5A).

#### 2.2.2. Signaling Related Genes

A wide range of signaling genes were differentially expressed. Many calcium signaling and receptor kinases were up and downregulated, including those with leucine-rich repeats. Genes related to sugar and nutrient signaling were also stimulated and upregulated. Genes encoding the mitogen-activated protein kinases (MAPKs) were upregulated due to the interaction with *H. seropedicae*.

Signaling proteins in signaling dependent on calcium, like calnexin and calreticulin appear down-accumulated, and one calmodulin was up-accumulated. The signaling G-proteins GTP-binding protein YPTM2 and Ras-related protein RABA2a are up-accumulated, while another Ras-related protein RABA2a appears down-accumulated. A protein that belongs to the 14-3-3 family also appears down-accumulated (Figure 5B).

#### 2.2.3. Protein Metabolism

A wide range of protein synthesis, modification, targeting, and degradation genes were expressed differentially. Overall, the genes related to the ubiquitin degradation process were expressed most differentially.

Protein metabolism included a higher number of differentially accumulated proteins, with 20 down-accumulated and 12 up-accumulated. Of these proteins, most were related to protein degradation, with seven up-accumulated and eight down-accumulated proteins, some of them related to degradation by the ubiquitin-proteasome complex, where the ubiquitin-activating enzyme E1 2 was up-accumulated, two subunits of the proteasome 26S up-accumulated, four subunits of the proteasome 26S down-accumulated, and one exclusively up-accumulated in the inoculated plants (Figure 5C).

#### 2.2.4. Transporters

A higher number of transcripts encoding transporters were stimulated by the interaction of the maize roots with *H. seropedicae* (FIGURE 5D). The transporters of amino acids were approximately 25% of the transporters expressed, with 30 transcripts, 15 being up and 15 downregulated.

ATP-binding cassette transporters (ABC transporters) were represented in the transporter genes expressed, with nine upregulated transcripts and 13 transcripts downregulated. Other transporters of phosphate and nucleotides had only upregulated transcripts. Sugar transporters were upregulated, with ten transcripts induced and six repressed in most of them.

A group of ten transcripts encoding the aquaporins were down and upregulated. On the other hand, two transcripts encoding ATPases were downregulated, and one was upregulated. In addition, the inoculation regulated five genes encoding proteins of nitrogen transporters with *H. seropedicae*, the nitrate transporter 2, high-affinity nitrate transporter and protein NRT1 were downregulated, while the nitrate transporter 1 and another protein NRT1 were upregulated (Figure 5D).

#### 2.2.5. Hormone Regulation

Plant interaction with *H. seropedicae,* stimulated the expression of hormone metabolism genes like IAA, abscisic acid (ABA), brassinosteroids, ethylene, salicylic acid and jasmonic acid cytokinin and gibberellin (Figure 5E). While auxin metabolism genes did not show, genes related to auxin responses were transcribed at a more significant level, the small auxin up RNAs (SAUR)-like auxin-responsive transcripts were the most induced. Genes encoding the IAA-amino acid synthetase GH3 conjugates the IAA with amino acids appear two times upregulated and one time downregulated, while the genes encoding IAA-amino acid hydrolases, and ILR1 appear downregulated.

Genes for biosynthesis of ABA involved in the cleavage of the precursor carotenoids zeaxanthin (ZEP) and the following step enzyme in the biosynthesis of nine-cis-epoxycarotenoid dioxygenase (NCED) were upregulated, and two other isoforms of proteins of NCED appear downregulated. In addition, the gene encoding the enzyme of the final step of production of ABA, aldehyde oxidase, was downregulated.

Gene encoding the 1-aminocyclopropane-1-carboxylate oxidase (ACC-oxidase), the final step of the biosynthesis of hormone ethylene, appears four times, three downregulated.

All the genes of the biosynthesis of gibberellin were downregulated, as well as the inactivation genes. However, a gene encoding gibberellin-regulated protein 1 was upregulated. On the other hand, the gene encoding the isopentenyl transferase1 (IPT) enzyme begins the biosynthesis of cytokinin and cytokinin oxidase, an enzyme that catalyzes the degradation irreversibly, cytokinin, was downregulated.

A few proteins related to hormone metabolism appear in the proteomic analysis. For example, only two isoforms of indole-3-acetaldehyde oxidase are responsible for the final conversion of indol-3-acetaldehyde to indol-3-acetic acid by the tryptamine pathway and SRG1 protein (senescence-related gene 1) related to ethylene appears up-accumulated (Figure 5E).

#### 2.2.6. Polyamine Metabolism

The polyamine (PA) metabolism appears repressed in transcript levels, as the genes S-adenosylmethionine decarboxylase (Samdc), arginine decarboxylase (Adc) and spermidine synthase (Spds) were downregulated, while at the proteomic level, the Spds are up-accumulated (Figure 5F).

#### 2.2.7. N and Amino Acid Metabolism

Inoculation with *H. seropedicae* induced N metabolism in the plants. As a result, the transcription of the enzyme nitrate reductase (NR) was upregulated, just like the enzyme glutamate synthase 1 and 2 (GOGAT). On the other hand, the enzyme glutamine synthetase 3 (GS) had its transcription repressed.

The inoculation stimulated a significant number of transcripts related to amino acid metabolism. Transcripts of enzymes with the aminotransferase function, alanine and aspartate, appear down and upregulated a few times. In addition, the enzyme glutamate decarboxylase (GAD) had transcription upregulated.

Genes encoding enzymes of the precursor and the tryptophan synthesis pathway, like chorismate synthase, anthranilate synthase, phosphoribosyl-anthranilate transferase, indole-3-glycerol phosphate synthase and tryptophan synthase appear downregulated almost all the time.

Proteins of N metabolism were all up-accumulated: the enzyme nitrate reductase, glutamine synthetase, and glutamate dehydrogenase. In addition, enzymes with aminotransferase function, such as alanine aminotransferase and aspartate aminotransferase/glutamate-oxalacetate transferase were all up-accumulated.

Amino acid metabolism had a higher number of proteins up-accumulated, with 16 proteins up-accumulated and only 3 down-accumulated. The group of up-accumulated proteins synthesize amino acids, including aspartate, alanine, threonine, serine-glycine-cysteine group and chorismate; and some proteins involved in the degradation of tryptophan and tyrosine (Figure 5G).

#### 2.2.8. Energy Production

Production of energy by the tricarboxylic acid cycle appears activated with genes that encode the enzymes of the cycle up and downregulated. For example, aconitase Succinate dehydrogenase and the enzyme pyruvate dehydrogenase were upregulated at the beginning of the cycle. On the other hand, the enzymes 2-oxoglutarate dehydrogenase and malate dehydrogenase were downregulated.

Proteins of the tricarboxylic acid cycle like aconitate hydratase, fumarate hydratase, isocitrate dehydrogenase and succinate-CoA ligase were shown to be up-accumulated (Figure 5H).

### 2.3. Validation of the DEGs and Proteins by Real-Time Quantitative PCR

To validate the reliability of gene expression and protein accumulation obtained by the transcriptomic and proteomic approaches, genes and proteins were validated by RT-qPCR. The internal control genes for α and β-tubulin showed constant levels of transcription between the control and inoculated samples according to the 2^−ΔΔCT^.

When compared with the expression analysis measured by RNA-Seq and the protein accumulation by proteomics, the DEGs and DAPs exhibited a similar regulation pattern under the inoculation treatment by RT-qPCR analysis.

These comparisons of data from RNA-Seq, proteomic and RT-qPCR analyses of the maize roots inoculated or not, with the plant growth-promoting bacteria *H. seropedicae* validated the findings of our transcriptomic and proteomic study (Table 2).

### 2.4. Phytohormone Analyses

To elucidate the effects of the inoculation of *H. seropedicae* on the hormonal balance of maize seedlings, samples of roots and shoots were prepared to quantify the hormonal contents. The results demonstrated that the seeds’ roots obtained higher auxins, gibberellins, cytokinin, ABA, brassinosteroids, and salicylic acid values.

In the shoots, the IAA was higher in the inoculated plants, but the other forms of the hormone-like IBA and 4-Cl-IAA did not show statistical differences between the inoculated and uninoculated plants. However, the contents of the gibberellins, cytokinins, ABA, brassinosteroids and salicylic acid were higher in the plants that were inoculated (Table 3).

## 3. Discussion

### 3.1. Maize Growth Promotion and Hormonal Crosstalk

*H. seropedicae* strain HRC54 (*Hs*-HRC54) cells, were higher in inoculated than in uninoculated roots (10^6^ to 10^2^). The presence of *Hs*-HRC54 in the maize roots at 5 d.a.i. shows the capacity of this microorganism to colonize the plant tissue and promotes an early effect of biostimulation. These results corroborate the counts of *H. seropedicae* strain SmR1 inoculated in maize plants [28] and explain the effects of biostimulation of the inoculation of *H. seropedicae* promoting the development of the roots and shoots. In other experiments, the inoculation with *H. seropedicae* exerted biostimulation of the growth of the maize root area when combined with humic substances after 7 d.a.i. [24].

Meantime, even though the inoculation promoted an increase in the biomass in the roots at 8 d.a.i., there were no statistically significant results in the analysis of scanned images of the roots in the parameters of length, projected and surface area, and root volume at 5 d.a.i. However, it was possible to observe that the roots of the inoculated seedlings had higher values than the control plants for the parameters, tips, forks and crossings, which may indicate the beginning of an increase in the number of lateral roots. Results with a biostimulation effect and increased lateral roots were obtained between 7 and 10 d.a.i. of *H. seropedicae* SmR1 in maize seedlings [29]. On the other hand, Hardoim et al. [30] demonstrated that the number of lateral roots of maize seedlings did not present differences between 3 d.a.i. with *H. seropedicae*, but only 7 d.a.i.

This effect of early biostimulation can be associated with phytohormones, such as auxins and gibberellins produced by *H. seropedicae* [15]. Results demonstrated biostimulation in wheat and rice seedlings, in terms of superficial root area and shoot and root length, through the production of IAA by *Herbaspirillum* spp. [17]. The capacity of *H. seropedicae* to influence plant hormonal status by the production or metabolism of hormones was supported by the detection of hormones in maize plant cells. The hormonal quantification demonstrated that inoculated plants obtained higher IAA, IBA and 4-Cl-IAA in the roots but only higher contents of IAA in the shoots. However, it was not possible to find transcripts related to the biosynthesis of auxin and only one enzyme involved in synthesis of this hormone up-accumulated with a 0.84 fold-change, but the gene expression was repressed by a −2.34 fold-change when analyzed by RT-qPCR. Similar results were obtained with the transcripts related to auxin [30].

Regulation of genes related to responses to the presence of auxin (SAUR) [31], genes that catalyze auxin conjugation and binding of free IAA to amino acids, conjugation to amino acids (GH3) [32] and the amidohydrolases ILR1, that hydrolyze the IAA-amino acid conjugates [33,34], demonstrate the effect of the inoculation on hormonal balance of the plant, where *H. seropedicae* can provide auxin to the plant. At the same time, it must promote hormone homeostasis. This can be related to the fact that the auxin provided by *H. seropedicae* activated the transduction signal, where auxin can signal for ubiquitin to bind to the repressor IAA/AUX, leaving it to be degraded by the ubiquitin-proteasomal complex. This releases the TF ARF to form a homodimer and initiate the expression of new IAA/AUX that will repress the expression of auxin synthesis genes by feedback regulation, and express genes in response to auxin, like SAUR, GH3 and ILRs [35,36]. Our results demonstrated that TFs IAA/AUX and genes and proteins of the ubiquitin-proteasome complex were differentially expressed by the inoculation of *H. seropedicae*, suggesting that auxin synthesis was under feedback regulation.

The same could be observed for the hormone gibberellin, where the hormonal quantification showed a higher content of the gibberellin forms GA1 and GA3 in the roots and shoots of the inoculated plants. At the same time, the transcriptomic analysis demonstrated that genes involved in the biosynthesis of this hormone appear almost all downregulated. These results can be interpreted as an effect of the production of gibberellin by *H. seropedicae* similar to that described by [15], which leads to feedback regulation since TFs (bHLH), gibberellins regulated proteins (GAST1), and gibberellin receptors (GID1L2) involved in the transduction pathway were differentially expressed after treatment with HRC54. Growth-promotion effects in plants by beneficial bacteria is known as phytostimulation. A crosstalk between the plant and bacteria that involves auxin has been demonstrated [37]. The auxin affects meristematic zones of tissues active in cell division and presents accelerated growth, while gibberellins can promote cell elongation and division. Gibberellin produced by PGPB can act in the early stages of plant development by improving the growth of the shoots and roots, and increasing root-hair density [14,16]. External application of gibberellin can induce the expression of tonoplast intrinsic protein (TIP), an aquaporin involved in cell elongation [38,39]. Our results demonstrated three genes encoding TIPs.

IAA in plants triggers the cell cycle to restart in the pericycle, establishing the lateral root’s mitotic sites [40], as described earlier in the results where WinRHIZO analysis showed the inoculation induced an increase in tips, forks and crossings in the roots. Furthermore, the emergence of lateral roots can promote the endophytic colonization of *H. seropedicae* into the xylem vessels [9,10,41]. This correlates with the results of cell quantification of *H. seropedicae* established in the roots from the inoculated plants, confirming the results of their growth promotion.

Results involving signaling genes and proteins presented here, showed the regulation of expression in response to the inoculation of *H. seropedicae* HRC54; therefore, these signaling components seem to play an essential role in signalizing the presence of the bacterial hormone for root growth. Plants can recognize cytoplasmatic Ca^2+^ alterations as a response signal to different stimuli. For example, CAMKs (calmodulin kinases) control the levels of Ca^2+^, have many isoforms, and can be differently expressed in response to cold, heat shock, light, touch stimulation, hormones such as auxin and oxidative stresses, some isoforms being specific to biotic stresses such as salicylic acid-mediated defense [42]. In addition, CAMKs interacting with Ca^2+^ (CCaMK) expression are observed in root-tip, and play a role in mitosis and meiosis [43]. The inoculation with the plant growth-promoter rhizobacterium *Azospirillum brasilense* in rice seedlings increased the number of lateral roots and the activity of calcium-dependent protein kinases (CDPK). The results of biochemical assays with a suppressor (W-7) of calmodulin (CaM), in rice roots demonstrated a reduction in the activity of CDPKs, and a dose-dependent decrease in the development of lateral roots, or disappearance of the root system [44]. The expression of CAMKs can be modulated by auxin [31]. Therefore, it can be associated with the inoculation of bacteria that produce this phytohormone. The SAURs are a group of early auxin-response genes in plants encoding short transcripts that accumulate rapidly, specifically when treated with auxin. CAMKs can bind to SAURs transcripts, modulating cell functions such as cell elongation [31]. GTP-binding and the Ras-related proteins control secretory vesicles on the vectorial flow of membrane material through a metabolic cascade to the surface in response to growth stimuli, such as auxin [45,46]. Also, there is an involvement in the 14-3-3 protein in plant hormone signaling regulation and homeostasis [47].

### 3.2. Modulation of the N and Amino Acids Metabolism

The diazotrophic bacterium *H. seropedicae,* can affect the nitrogen metabolism of the plant host [25,26,48,49,50]. Using transcriptomic and proteomic approaches, this study allowed us to better understand how inoculation can modulate nitrogen acquisition and metabolism. The genes encoding high-affinity nitrate transporter proteins (NRT1 and NRT2.2) appear upregulated, while other nitrate transporters were downregulated, indicating a regulation of the absorption of this nutrient. Similar profiles in the transcription of nitrate transporters were demonstrated by the inoculation of *H. seropedicae* HRC54 in the maize inbred line UENF 506–8 [30]. Expression of nitrate transporters can demonstrate the capacity of the diazotrophic bacterium to provide N to the roots, improving the uptake of this nutrient in the plant. For example, the results of the inoculation of *H. seropedicae* promoted an increase of 120% in the N content in rice plants [12].

Nitrate absorbed by the roots is reduced to nitrite in the cytosol by the enzyme nitrate reductase (NR) and transported to the plastids and converted to ammonia by the enzyme nitrite reductase (NIR). Ammonia can then be incorporated into an amino acid by the enzymes glutamine synthetase (GS) and glutamine-2-oxoglutarate aminotransferase (GOGAT) or glutamate synthase, generating glutamine (GLN) and glutamate (GLU), respectively [51]. There are two forms of GOGAT enzyme; one receives electrons from NADH and the other from ferredoxin (Fd). The NADH-GOGAT is in the non-photosynthetic plastids, such as in root cells and vascular bundles and is involved in NH_4_^+^ assimilation from the rhizosphere. On the other hand, the Fd-GOGAT is present in the root’s plastids by NO_3_^−^ nutrition and is responsible for assimilating glutamine generated by the NO_3_^−^. The increase in transcript and proteins levels of the NR, the GS and GOGAT enzymes and the ferredoxin in the plastids in this study allow us to say that inoculated maize plants were able to assimilate the nitrate provided by the diazotrophic bacterium and incorporate them in GLN and GLU, corroborating results that demonstrated higher NR activity in maize and rice [24,48,52] and GLN and GLU contents in rice plants inoculated with *H. seropedicae* [52].

GDH catalyzes the reversible reaction of synthesis or deamination of glutamate to α-ketoglutarate in the presence of NADH or NADPH and NH_4_^+^, serving as a carbon skeleton source for building amino acids or introducing carbon into the tricarboxylic acid (TCA) cycle in the form of 2-oxoglutarate [53,54]. The primary physiological function of NADH-GDH was linked to TCA, providing 2-oxoglutarate to form amino acids, leading to the accumulation of alanine, gamma-aminobutyrate (GABA) and aspartate in the roots and leaves [55]. GLU can also be metabolized to GABA via the GAD enzyme and converted into succinic semialdehyde, connecting the C/N with the PA pathway [56]. Polyamines are low-molecular-weight polycations with participation in growth and physiological processes, forming GLU metabolism leading to the formation of the PAs putrescine (Put), spermidine (Spd) and spermine (Spm), which are an essential group of reactions for the interaction of carbon (C) and nitrogen (N) connecting the urea pathway to TCA [56,57,58]. The aminotransferase enzymes play an essential role in transamination reactions, incorporating N into other amino acids. The aspartate aminotransferase (Asp-AT) catalyzes the reaction where the GLU + oxaloacetate is converted to aspartate + 2-oxoglutarate. The aspartate participates in the transport of malate–aspartate for the TCA and the process of carbon fixation in the C4 plants [40]. The alanine-aminotransferase (Ala-AT) catalyzes the reversible transfer of the amino group from the alanine to 2-oxoglutarate to form pyruvate and glutamate [59].

**Figure 6:**
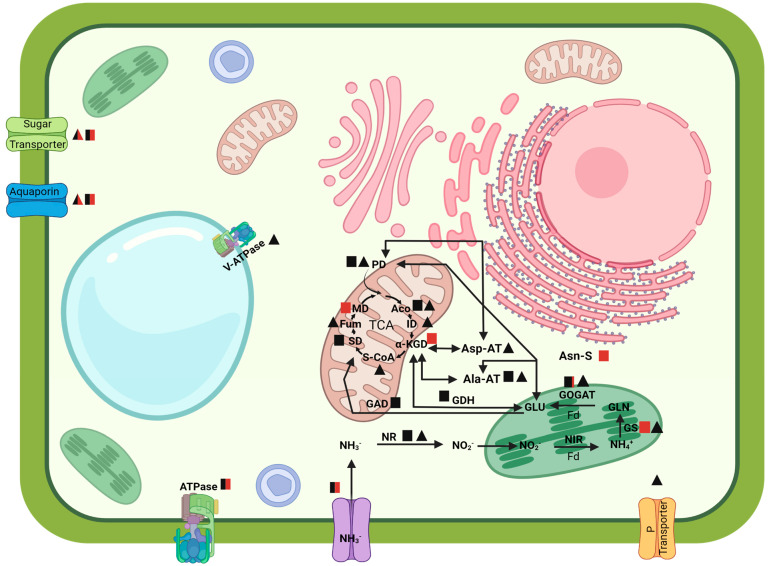
Integration of transcripts and proteins related to N uptake and assimilation and the interaction with the TCA. ■ Transcripts (black = upregulated; red = downregulated; black/red= transcript up and downregulated), ▲ Proteins (black = up-accumulated; red = down-accumulated; black/red = proteins up and down accumulated). TCA: tricarboxylic acid cycle; Aco: anonitase; ID: isocitrate dehydrogenase; α-KGD: α-ketoglutarate dehydrogenase; S-CoA: succinyl-CoA synthetase; SD: succinate dehydrogenase; Fum: fumarase; MD: malate dehydrogenase; PD: pyruvate dehydrogenase; GDH: glutamate dehydrogenase; GAD: glutamate decarboxylase GLU: glutamate; GOGAT: glutamate synthase; GLN: glutamine; GS: glutamine synthetase; Fd: ferredoxin; NH4+: ammonia; NIR: nitrite reductase; NO_2_: nitrite; NR: nitrate reductase; NO3-: nitrate; Asn-S: asparagine synthase; ASP-AT: aspartate aminotransferase; Ala-AT: alanine aminotransferase; Sugar: sugar transports; Aqua: aquaporins; P: phosphate transporter.

The presented results demonstrate a balance in the C/N ratio through the crosstalk of N metabolism and the TCA. The enzymes GDH, Asp-AT and Ala-AT appear up-accumulated in the proteomic analysis, suggesting that N coming from the N fixation by the inoculation of *H. seropedicae,* in addition to being assimilated in the form of glutamate, was incorporated into other amino acids, and these were able to supply carbon to TCA, integrating the metabolic pathways. Our results demonstrated that the genes and enzymes of the TCA were induced and repressed by the inoculation with *H. seropedicae*, indicating the relationship between the two metabolic pathways. Studies of the metabolome of maize plants show that roots inoculated with *H. seropedicae* exude organic acids originating from the TCA [60]. The scheme in Figure 6 represents the integration of the data obtained by this study related to N uptake and assimilation in maize roots by the effect of inoculation of *H. seropedicae* HRC54.

Chlorophyll content in the leaf can be used to predict the nutritional level of nitrogen in plants because the amount of this pigment correlates positively with N content [61]. The photosynthetic analyses and the expression of the CAB-1 gene, a protein that makes up the complex photosystem light collector, where approximately 60% of all plant chlorophyll is bound [62] also reflect the initial N uptake and assimilation increase, with a slight difference in the chlorophyll content. Other works with *H. seropedicae* in maize plants with late stage analyses demonstrated that inoculation could increase the chlorophyll content and photosynthetic rates [12,23,24].

This study attempts to establish expression trends of genes and protein functional groups in Maize inoculated with *H. seropedicae* strain HRC54. RNA-seq and differential proteomics were employed to quantify changes during the early stages of HRC54-treated plant development. This data was compared with plant growth and physiological parameters. The integration of transcriptome and proteome data of maize roots inoculated or not with *H. seropedicae* demonstrated a regulation at the transcriptional and post-transcriptional levels that reflect the protein quantity, generating a low correlation between the results of the approaches, but show regulation at two levels. This is a typical event when comparing omics results because mRNA and protein levels do not necessarily correlate, according to many studies of the integration of transcriptome and proteome in maize plants [63,64,65].

## 4. Materials and Methods

### 4.1. Preparation of Inoculum

The microorganism used was *H. seropedicae* strain HRC54 (SisGen n° AFD1CAD), originally isolated from sugarcane roots. The pre-inoculum was obtained from a pure plate colony and after growth in DYGS [66] liquid medium for 24h at 30 °C in an orbital shaker at 150 rpm. After growth, a 20 μL aliquot of the bacterial suspension was transferred to JNFB [66] liquid medium supplemented with NH_4_Cl (1g.L^−1^) for 48 h under the same conditions described above. After 48 h of growth, the bacterial cells were sedimented by centrifugation (5.000× *g* for 15 min) and resuspended in sterile distilled water at a cell density of 4 × 10^9^ CFU.mL^−1^.

### 4.2. Plant inoculation, Weight Measures and Root Morphology

Maize seeds were used (*Zea mays* L., var. Dekalb 7815). The seeds were surface disinfected by immersion in 0.5% NaClO for 5 min, followed by rinsing and immersion in distilled water for 6 h. Afterwards, the seeds were placed to germinate on Germitex paper for 72 h. Seeds with radicles approximately 2.5 cm long were selected and placed in 2 L pots filled with 2 mM CaCl_2_ solution, making 40 seeds per pot. Maize seedlings were inoculated with a 20 mL aliquot of the bacterial inoculum. Tests were carried out under the following conditions: uninoculated maize plants (Control) and inoculated maize plants, with 2x10^7^ cells mL^−1^ *H. seropedicae* strain HRC54 at 28 °C, photoperiod of 16h/8h and aeration by an aquarium pump. After five days of biostimulation, the plants were collected and separated into biological triplicates to determine the fresh and dry weight of the shoots and the roots. Next, roots were separated for the extraction of RNA and DNA.

Five fresh roots of control and inoculated plants were scanned by a printer (Epson Expression 10000xL 1.8 v3.49) to analyze the root system morphology to obtain images in the WinRhizo^®^ software (Regent Instruments Inc., Quebec, Canada). Analyzed parameters were: length, projected area, surface area, root volume, number of tips, and number of forks and crossings.

The green index was measured three times per leaf from five plants via the SPAD-502 Plus chlorophyll meter (Konica Minolta, Tokyo, Japan), indicating the amount of chlorophyll in the leaf tissue.

Data were analyzed by two-way statistical analysis of variance (ANOVA) followed by a Tukey’s test. Data analyses were carried out using GraphPad Prism 7.00 (https://www.graphpad.com, accessed on 15 October 2022). In all cases, the differences were considered significant at *p* < 0.05.

### 4.3. Quantification of Diazotrophic Bacteria and H. seropedicae in Maize Roots

To quantify total diazotrophic bacteria associated with the maize roots, these were washed with sterile water and pounded in a sterile mortar with a saline solution of 0.9%. Serial dilutions of 10^−3^ to 10^−7^ were made and inoculated in semisolid, N-free culture media JNFb. Diazotrophic bacteria populations were counted based on the most probable number (MPN) using the McCrady table for three replications per dilution [66]. To quantify *H. seropedicae* stabilized in the inoculated and uninoculated maize roots, total DNA was isolated using DNAzol (Thermo Fischer) as described in the manufacturer’s protocol. Total DNA samples were quantified using a NanoDrop ND-1000 spectrophotometer and were standardized to the final concentration of 40 ng.μL^−1^. Specific primers for *H. seropedicae* ribosomal DNA 16S (16S rDNA) were used (5′-CTAATACCGCATACGATCTAC-3’ and 5′-TTCTGGATATTAGCCAAAACC-3’) for PCR amplification. The protocol consisted of an initial incubation at 50 °C for 2 min, 95 °C incubation for 10 min, followed by 40 cycles of 95 °C for 15 s and 60 °C for 1 min [67]. This PCR aimed to detect the presence of the bacteria in the extracted material from three control and three inoculated plants.

### 4.4. RNA Extraction and Sequencing

100 mg of control and inoculated roots were macerated in liquid nitrogen for RNA extraction. According to the manufacturer’s instructions, the total RNA of the samples was extracted with the RNeasy Plant Mini Kit (Qiagen, São Paulo, Brazil). Total RNA was quantified using the NanoDrop ND-1000 spectrophotometer. RNA was eluted in DEPC-treated water (total amount of 4–10 μg RNA), digested with DNAse, and depleted of ribosomal RNA using the GOTAQ^®^ 1-STEP RT-qPCR (PROMEGA). Subsequently, a 1% RNAse free, agarose gel was made to analyze the extracted RNA. According to the manufacturer’s protocol, sequencing libraries were prepared using the Whole Transcriptome Analysis kit (Applied Biosystems). Libraries were sequenced on the Illumina platform by Lactad company—in Brazil.

### 4.5. Bioinformatic Analysis of the Sequences Obtained by RNA-seq

We used FastQC to assess read quality and remove low-quality ones [68]. To remove possible residual rRNA contamination, the filtered reads were aligned to *Z. mays* rRNA sequences (GenBank: AH001709.2, AH001710.2) using BLASTN and Novoalign (http://www.novocraft.com, accessed on 14 July 2019) with default parameters. The resulting RNA-seq reads were mapped to the maize reference genome version 4 (RefGen.B73.v4) using STAR [69]. Read counts per gene for uniquely mapped reads were estimated with feature Counts v2.0.0 [70]. Correlations between biological replicates were calculated with R’s core function (http://www.r-project.org/, accessed on 14 July 2019). Genes with mean counts ≥ 1 were considered expressed.

Differential expression analysis for inoculated versus control with *H. seropedicae* was performed in the R packages edgeR [71] and DESeq2 [72], and the genes with FDR ≤ 0.05 were considered differentially expressed. Only the differentially expressed genes (DEGs) identified by both methods were used in further analyses to reduce type I errors. Expression estimates are represented as DESeq normalized values. Metabolic classification analysis was executed with MapMan version 3.6.0RC1 [73,74] (https://mapman.gabipd.org/, accessed on 3 May 2020).

### 4.6. Proteomic Procedures

#### 4.6.1. Protein Extraction

Three biological replicates (300 mg of root material for each replicate from a mixture of five individual plantlets) of the treatment control and inoculated after five days of growth were used for protein extraction. Proteins were extracted using the trichloroacetic/acetone precipitation method [75]. Three biological samples from each treatment were resuspended in 1 mL of chilled extraction buffer containing 10% (*w*/*v*) trichloroacetic acid (TFA; Sigma Chemical Co., St. Louis, MO, USA) in acetone with 20 mM dithiothreitol (DTT; GE Healthcare), vortexed for 30 min at 8 °C and left at −20 °C for 1 h for protein precipitation. The mixture was centrifuged at 16,000× *g* for 30 min at 4 °C. The resulting pellets were washed three times with cold acetone + 20 mM DTT, vortexed for 30 s and centrifuged for 5 min at 4 °C for each wash. Finally, pellets were air-dried and resuspended in 1 mL of buffer containing 7 M urea (GE Healthcare, Little Chalfont, UK), 2 M thiourea (GE Healthcare), 2% Triton X-100 (GE Healthcare), 1% DTT, 1 mM phenylmethylsulfonyl fluoride (PMSF; Sigma-Aldrich), and complete protease inhibitor cocktail (Roche Diagnostics, Mannheim, Germany) and incubated for 30 min on ice. Samples were then vortexed at 8 °C for 30 min and centrifuged at 16,000× *g* for 20 min at 4 °C. The supernatant was collected, and the protein concentrations were determined using the 2-D Quant Kit (GE Healthcare, Piscataway, NJ, USA).

#### 4.6.2. Protein Digestion

For protein digestion, 100 µg of extracted proteins from each biological replicate were precipitated with methanol/chloroform [76] to remove any interference from the samples. After the protein precipitation, the samples were resuspended in 7 M urea/2 M thiourea solution for proper suspension. Tryptic protein digestion (1:100 enzyme: protein, V5111, Promega, Madison, USA) was subsequently performed using the modified filter-aided sample preparation (FASP) method [77]. The resulting peptides were quantified according to the A205 nm protein and peptide method using a NanoDrop 2000c spectrophotometer (Thermo Fisher Scientific, Waltham, MA, USA).

#### 4.6.3. Mass Spectrometry Analyses

Nano-LC-electrospray ionization (ESI)-MS/MS analysis was carried out using a nanoAcquity UPLC (Waters) coupled to a Synapt G2-Si mass spectrometer (Waters). The peptide mixtures were separated by liquid chromatography by loading 1 μg of digested peptides onto a nanoAcquity UPLC 5-μm C18 trap column (180 μm by 20 mm; Waters), followed by loading onto a nanoAcquity HSS T3 1.8-μm analytical column (75 μm by 150 mm; Waters) at a rate of 400 nL.min^−1^ at 45 °C [78].

#### 4.6.4. Proteomics Data Analysis

Spectral processing and database searching was performed using ProteinLynx Global Server (PLGS; v3.0.2; Waters) and ISOQuant workflow software [79]. The *Z. mays* protein database from Uniprot (Proteome ID: UP000007305) was used for protein identification. Label-free relative quantitative analyses were performed based on the normalized protein ion counts. The spectra processing in PLGS and comparative label-free quantification analysis parameters in ISOQuant were described by [77]. After ISOQuant data analyses, only present or absent proteins (for unique proteins) in all three biological replicates were considered for differential abundance analysis. Data were analyzed using Student’s *t*-test (two-tailed). Proteins with *p*-values of *p* ≤ 0.05 were considered up-accumulated if the |log2 fold change| was more significant than 0.5 and downregulated if the |log2 fold change| was lower than −0.5.

Finally, proteins were blasted against the non-redundant (nr) Plants/ Viridiplantae_Protein_Sequences database by using the Blast2GO software (www.blast2go.com, accessed on 3 May 2020) [80]. Metabolic classifications were executed with MapMan version 3.6.0RC1 [73,74] (https://mapman.gabipd.org/, accessed on 3 May 2020). A Venn diagram was constructed as described [81] (http://bioinfogp.cnb.csic.es/tools/venny/index.html, accessed on 3 May 2020), and was used to analyze the correlation of common DEGs and DAPs in the treatments.

### 4.7. Validation of DEGs and Proteins by Real-Time Quantitative PCR

A new set of RNA samples of roots and leaves were prepared with Trizol^®^ reagent (Thermo Fisher Scientifi, Waltham, MA, USA), and the cDNA synthesis was carried out using the ImProm-IITM kit (Promega) as described in the manufacturer’s protocol using 1µg from RNA extracted from the inoculated and uninoculated samples.

Real-time PCR quantification validated the differentially expressed genes identified through transcriptome and proteomic analyses. Specific primers for each gene to be analyzed (Table S1) were designed using Primer3 Plus (https://primer3plus.com/, accessed on 14 October 2022). Genes for α-Tubulin (α-Tub) and Tubulin β-chain (β-Tub) were used as normalizing genes [82].

Primers were used in RT-qPCR reactions at concentrations of 500 nM, using 7.5 µL of FAST SYBR Green PCR Master Mix (Applied Biosystems, Waltham, MA, USA) in a Step One Plus Real-Time PCR machine (Applied Biosystems) under the following conditions: 40 cycles 95 °C for 1 min, 60 °C for 1 min and 72 °C for 1 min. All qPCR runs were conducted in triplicate. The relative quantification was determined using the 2^−∆∆∆Ct^ method [83].

### 4.8. Phytohormone Analyses

Phytohormones levels were determined using high-performance liquid chromatography (Agilent 1100 series HPLC system), including an autosampler, a quaternary pump, a degasser, and a fluorescence detector. Approximately 50–100 mg of fresh leaves were sealed in 1.5 mL snap-cap vials. After being frozen in liquid nitrogen, the leaves were ground into powder, and 500 µL of 1-propanol/H_2_O/concentrated HCl (2:1:0.002, vol/vol/vol) with internal standards (10–50 ng) were added, followed by agitation for 30 min at 4 °C. CH_2_Cl_2_ (1 mL) was then added, followed by agitation for another 30 min and centrifugation at 13,000× *g* for 5 min [84]. After centrifugation, two phases were formed, and plant debris was in the middle of the two layers. The lower layer (around 1 mL) was concentrated and re-solubilized in methanol (0.3 mL), of which 25 µL was injected into the column for analysis. The lower layer (25 µL) was also directly injected into a column for analysis. Phytohormones were separated by an HPLC equipped with a reversed-phase column (C18 Gemini 5µ, 150 × 2.00 mm, Phenomenex, CA, USA) using a binary solvent system composed of water with 0.1% HCO_2_H (A) and methanol, with 0.1% HCO_2_H (B) as a mobile phase at a flow rate of 0.3 mL.min^−1^. Separations were performed using a gradient of increasing methanol content. The initial gradient of methanol was maintained at 30% for 2 min and increased linearly to 100% at 20 min, and the fluorescence detector was set with the excitation wavelength (Ex) of 230 nm and the emission wavelength (Em) of 360 nm. The injection volume was 5 μL for each analysis [85]. Data are expressed as means ± SEM and were analyzed by two-way statistical analysis of variance (ANOVA) followed by a Tukey’s test. All treatments were performed in quintuplicate. Data analyses were carried out using GraphPad Prism 7.00 (https://www.graphpad.com, accessed on 14 October 2022). In all cases, the differences were considered significant at *p* < 0.05.

## 5. Conclusions

The data obtained in this work reveal molecular mechanisms in the interaction between plant bacteria and, consequently, the physiological mechanisms involved in promoting the growth of maize plants by *H. seropedicae*. The multi-omics data integration employed in this study shows the induction of different pathways by *H. seropedicae* in roots of maize plants, suggesting the promotion of growth in the early stages of development arose from modulation of N uptake and assimilation, plus regulation and phytohormone signaling. The work results contribute to understanding the molecular mechanisms regulated by beneficial bacteria to promote maize plant growth.

## Figures and Tables

**Table 1:** Summary of RNA-seq data of the roots inoculated or uninoculated with *H. seropedicae* strain HRC54.

Samples	Uniquely Mapped	Mapped to Multiple Loci	Mapped to too Many Loci	Unmapped: Too Short	Unmapped: Other	% of Mapped Genes
Control 1	76,454,565	4,169,270	462,100	6,955,351	35,217	86.80
Control 2	27,685,993	1,565,491	55,449	2,497,993	19,093	86.99
Control 3	51,901,676	2,850,385	260,875	4,381,316	29,724	87.34
Inoculated 1	34,434,572	2,451,679	754,582	14,461,553	36,493	66.04
Inoculated 2	13,506,360	806,095	27,396	1,936,178	13,038	82.91
Inoculated 3	33,860,470	2,281,374	324,017	10,106,497	32,632	72.65

**Table 2:** Genes and proteins validated by the RT-qPCR.

	RNA-Seq	Proteomic	RT-qPCR
*b6f*	1.88	-	5.41
*CALM1*	-	1.62	4.55
*GID1L2*	-	0.6	4.08
*PsbO_1*	0.14	-	3.79
*GAST1*	0.03	-	3.39
*PHTP9*	0.15	-	2.40
*CAB-1*	2.12	-	2.25
*PIP1-5*	1.01	−1.1	2.02
*AO2*	-	0.84	−2.34
*CHOR*	-	0.68	0.17
*HMTD*	−0.63	-	−0.40

The expression values in this table are in Log² fold-change.

**Table 3:** Quantifying phytohormones (ng/mL) in maize plant controls and those inoculated with *H. seropedicae*.

	Root		Shoots	
	Uninoculated	Inoculated	Uninoculated	Inoculated
IAA	15.49 ± 0.41 B	29.63 ± 0.51 A	15.34 ± 0.39 B	19.57 ± 0.37 A
IBA	11.13 ± 0.72 B	14.62 ± 0.24 A	11.37 ± 0.43 B	12.08 ± 0.17 B
4-Cl-IAA	4.3 ± 0.35 B	8.67 ± 0.33 A	4.35 ± 0.35 B	4.67 ± 0,33 B
GA_1_	8.73 ± 0.35 B	11.91 ± 0.19 A	9.045 ± 0.05 B	16.49 ± 0.29 A
GA_3_	14.84 ± 0.16 B	20.46 ± 0.29 A	14.67 ± 0.37 B	29.02 ± 1.14 A
T-ZEATIN	8.46 ± 0.41 B	10.56 ± 0.26 A	8.55 ± 0.25 B	14.27 ± 0.38 A
ABA	18.42 ± 0.44 B	21.41 ± 0.40 A	19.42 ± 0.21 B	25.35 ± 0.27 A
Brassinosteroids	3.22 ± 0.14 B	5.26 ± 0.50 A	4.42 ± 0.25 B	7.72 ± 0.24 A
Salicylic acid	1.70 ± 0.26 B	5.39 ± 0.21 A	2.68 ± 0.21 B	2.59 ± 0.50 B

(±) represent the standard deviation (n = 5). Averages followed by identical capital letters do not differ in the inoculation condition (*p* value < 0.0001).

## Data Availability

The mass spectrometry proteomics data have been deposited to the ProteomeXchange Consortium via the PRIDE [1] partner repository with the dataset identifier PXD038609.

## Supplementary Materials

The following supporting information can be downloaded at: https://www.mdpi.com/article/10.3390/plants12010048/s1, Table S1: RNA-seq data of differentially expressed genes of maize roots inoculated with *Herbaspirillum seropedicae*; Table S2: Proteomic data of differentially accumulated proteins of maize roots inoculated with *Herbaspirillum seropedicae*; Table S3: primers used in the RT-qPCR validation.
